# The protocols for the 10/66 dementia research group population-based research programme

**DOI:** 10.1186/1471-2458-7-165

**Published:** 2007-07-20

**Authors:** Martin Prince, Cleusa P Ferri, Daisy Acosta, Emiliano Albanese, Raul Arizaga, Michael Dewey, Svetlana I Gavrilova, Mariella Guerra, Yueqin Huang, KS Jacob, ES Krishnamoorthy, Paul McKeigue, Juan Llibre Rodriguez, Aquiles Salas, Ana Luisa Sosa, Renata MM Sousa, Robert Stewart, Richard Uwakwe

**Affiliations:** 1 King's College London, Health Service and Population Research Department, Section of Epidemiology, De Crespigny Park, SE5 8AF, London, UK; 2 Internal Medicine Department, Geriatric Section Universidad Nacional Pedro Henriquez Ureña (UNPHU), John F Kennedy Avenue, Santo Domingo, Dominican Republic; 3 Behavioral and Cognitive Neurology Unit, Neuraxis Institute – Neurological Foundation, Buenos Aires – Argentina; 4 Mental Health Research Centre Russian Academy of Medical Sciences, Moscow, Russia; 5 Psychogeriatric Unit, National Institute of Mental Health "Honorio Delgado Hideyo Noguchi", Lima – Perú; 6 Institute of Mental Health; Peking University, # 51 Hua Yuan Bei Road Haidian District Beijing, 100083, China; 7 Christian Medical College, Vellore, India; 8 Srinivasan Centre for Clinical Neurosciences. The Institute of Neurological Sciences, Voluntary Health Services, Taramani, Chennai, India; 9 Genetics & Epidemiology Department Conway Institute – University College Dublin, Belfield Campus Belfield, Dublin, Ireland; 10 Facultad de Medicina Finley-Albarran, Medical University of Havana, Cuba; 11 Medicine Department, Caracas University Hospital, Faculty of Medicine, Universidad Central de Venezuela, Caracas; 12 The Cognition and Behavior Unit, National Institute of Neurology and Neurosurgery of Mexico, Av. Insurgentes # 3877. Col. La Fama. ZIP Code 14269. Delegacion Tlalpan. Mexico City, Mexico; 13 Dept. of Mental Health, Nnamdi Azikiwe University Teaching Hospital, Nnewi, Anambra State, NIGERIA

## Abstract

**Background:**

Latin America, China and India are experiencing unprecedentedly rapid demographic ageing with an increasing number of people with dementia. The 10/66 Dementia Research Group's title refers to the 66% of people with dementia that live in developing countries and the less than one tenth of population-based research carried out in those settings. This paper describes the protocols for the 10/66 population-based and intervention studies that aim to redress this imbalance.

**Methods/design:**

Cross-sectional comprehensive one phase surveys have been conducted of all residents aged 65 and over of geographically defined catchment areas in ten low and middle income countries (India, China, Nigeria, Cuba, Dominican Republic, Brazil, Venezuela, Mexico, Peru and Argentina), with a sample size of between 1000 and 3000 (generally 2000). Each of the studies uses the same core minimum data set with cross-culturally validated assessments (dementia diagnosis and subtypes, mental disorders, physical health, anthropometry, demographics, extensive non communicable disease risk factor questionnaires, disability/functioning, health service utilisation, care arrangements and caregiver strain). Nested within the population based studies is a randomised controlled trial of a caregiver intervention for people with dementia and their families (ISRCTN41039907; ISRCTN41062011; ISRCTN95135433; ISRCTN66355402; ISRCTN93378627; ISRCTN94921815). A follow up of 2.5 to 3.5 years will be conducted in 7 countries (China, Cuba, Dominican Republic, Venezuela, Mexico, Peru and Argentina) to assess risk factors for incident dementia, stroke and all cause and cause-specific mortality; verbal autopsy will be used to identify causes of death.

**Discussion:**

The 10/66 DRG baseline population-based studies are nearly complete. The incidence phase will be completed in 2009. All investigators are committed to establish an anonymised file sharing archive with monitored public access. Our aim is to create an evidence base to empower advocacy, raise awareness about dementia, and ensure that the health and social care needs of older people are anticipated and met.

## Background

### The 10/66 dementia research group

The title of the 10/66 Dementia Research Group [1-4] refers to the 66% of people with dementia that live in developing countries and the less than one tenth of population-based research carried out in those settings. We have attempted to redress the research imbalance through south-south and south-north research collaborations. Our first symposium in Cochin in 1998 established clear priorities [1], which have guided our subsequent research programme.

**1) Methodological problems needed first to be addressed**, particularly development of culture- and -education fair dementia diagnostic procedures, a prerequisite for meaningful comparison between regions [2]. Our pilot studies in 26 centres in Latin America and the Caribbean, Africa, India, Russia, China and SE Asia demonstrated the feasibility and validity of a one stage culture and education-fair diagnostic protocol for population-based research [5,6], the resulting '10/66 Dementia' outcome being derived from a probabilistic algorithm.

**2) More research was needed to describe dementia prevalence and incidence**. Local evidence of numbers of people affected, and associated burden would raise awareness and inform policymaking. Regional variations in disease frequency could give clues regarding aetiology, if obtained using harmonised and cross-culturally valid methods.

**3) Description of care arrangements for people with dementia**. In our pilot studies we showed that levels of caregiver strain, among caregivers of people with dementia are at least as high as those typically reported from developed countries, despite the strong traditions of extended family care in most of the countries studied [7-9]. We have also highlighted the lack of awareness and understanding of the nature of the dementia syndrome, and the unresponsiveness of health care services as currently constituted [8,10,11]. Behavioural symptoms, such as agitation, wandering, calling out repeatedly, are common in developing countries, associated with stigma and blame, and an important source of distress for caregivers [12].

**4) The effectiveness of new services for people with dementia and their caregivers**. We have shown that community health workers in Brazil [13] and India [14] can, after one day of training, identify cases of dementia in the community with reasonable accuracy using their knowledge of older people from their outreach work (which focuses mainly on maternal and child health). We have gone on to develop a simple five session caregiver education and training intervention, suitable for administration by a community health worker.

Since the pilot phase (1999–2001), the 10/66 DRG has conducted population-based surveys of dementia prevalence and impact in 14 catchment areas in 10 low and middle income countries (2003–2007). Nested within the population-based studies is a randomized controlled trial of a caregiver intervention for people with dementia and their families. We will shortly embark on an incidence phase with a 2.5 to three year follow-up of baseline participants in seven of the 10 countries (2007–2010). The purpose of this paper is to describe the protocols for the three elements of the research programme; the baseline survey, the incidence phase, the casefinder approach and the randomized controlled trial; and in doing so, publicize the publicly accessible 10/66 data archive that will be established by the following centres: India (Vellore and Chennai), Nigeria, China, Cuba, the Dominican Republic, Venezuela, Mexico, Peru and Argentina. The Brazilian centre will establish an independent data archive.

### The demographic and health transitions

**Figure 1:**
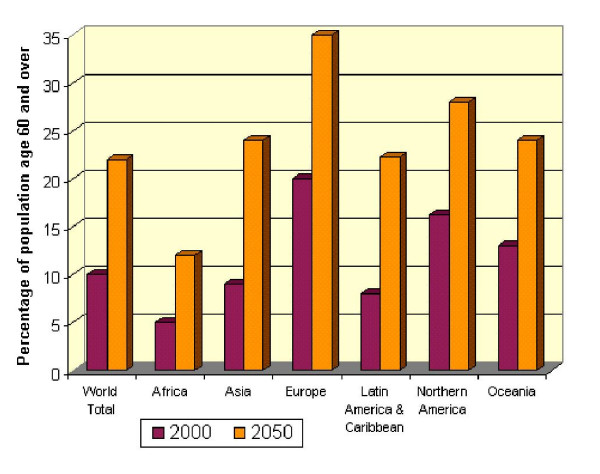
Global demographic ageing, 2000–2050 (UNDIESA).

**Figure 2:**
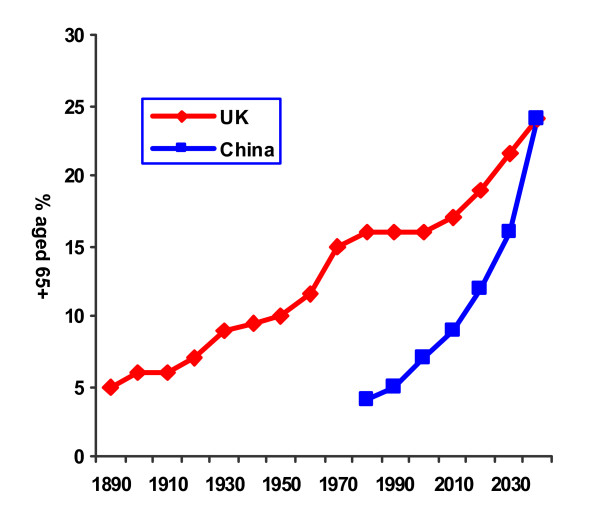
Demographic transition, UK and China.

Demographic ageing proceeds apace in all world regions, more rapidly than first anticipated [15]. The proportion of older people increases as mortality falls and life expectancy increases. Population growth slows as fertility declines to replacement levels. Latin America, China and India are experiencing unprecedentedly rapid demographic ageing (Figures 1 and 2).

In the accompanying health transition non-communicable diseases (NCD) assume a progressively greater significance in low and middle income countries (LAMIC). NCDs are already the leading cause of death in all world regions apart from sub-Saharan Africa. Of the 35 million deaths in 2005 from NCDs, 80% will have been in LAMIC [16]. This is partly because most of the world's older people live in these regions – 60% now rising to 80% by 2050. However, changing patterns of risk exposure also contribute. Latin America exemplifies the third stage of health transition. As life expectancy improves, and high fat diets, cigarette smoking and sedentary lifestyles become more common, so CVDs have maximum public health salience – more so than in stage 2 regions (China and India) where risk exposure is not yet so elevated, and in stage 4 regions (Europe) where public health measures reduced exposure levels [17]. The INTERHEART cross-national case-control study suggests that risk factors for myocardial infarction operate equivalently in all world regions, including Latin America and China [18].

### Dementia in developing countries

#### Global burden

It has been recently estimated that 24.2 million people live with dementia worldwide (based upon systematic review of prevalence data and expert consensus), with 4.6 million new cases annually [19] (similar to the annual global incidence of non-fatal stroke [20]). Most people with dementia live in LAMIC, 60% in 2001 rising to 71% by 2040. Numbers will double every twenty years to over 80 millions by 2040. Increases to 2040 will be much sharper in developing (300%) than developed regions (100%). Growth in Latin America will exceed that in any other world region. Well designed epidemiological research can generate awareness, inform policy, and encourage service development. However, such evidence is lacking in many world regions, and patchy in others, with few studies and widely varying estimates [19]. There is a particular dearth of published epidemiological studies in Latin America with two descriptive studies only, from Brazil [21,22] and Colombia[23].

#### Developed/developing country differences

Prevalence of dementia is somewhat lower in developing countries than in the developed north [2], strikingly so in some studies [24,25]. Our expert consensus panel, reviewing all available evidence, confirmed this trend [19], which seems not to be explained by differences in survival[25,26]. Mild dementia may be under-detected because of difficulties in establishing the criterion of social impairment. Differences in levels of exposure to environmental risk factors may also have contributed, with low levels of cardiovascular risk [27] and hypolipidaemia [28,29] in some developing countries suggested as explanations. Other potential risk exposures will be more prevalent in LAMIC, for example anaemia associated with AD in rural India [30]. Dietary deficiencies, particularly of micronutrients, are widespread and strongly linked to poverty. Deficiencies of folate and vitamin B_12 _are of particular interest given their consequences; anaemia, neuropathy, hyperhomocysteinaemia [31], increased risk of stroke and IHD [32]. Vitamin B_12 _deficiency is strikingly prevalent (> 40%) across Latin America [33-35], linked to gastrointestinal infections and diets deficient in meat and dairy produce [33]. Folate deficiency is endemic in those living in poverty [34], and after economic crisis [35]. Diets deficient in legumes may have contributed. Micronutrient deficiency is probably more prevalent in the elderly but there are few data on this age group [33]. Iodine deficiency has also been a major public health problem in most Latin American countries [36]. Iodized salt is now generally available but iodine content is poorly regulated [36]. We found only one study from the region, reporting a prevalence of sub-clinical hypothyroidism of 16.1% in post-menopausal Brazilian women [37]. High infant mortality may contribute to population differences in dementia frequency; constitutional and genetic factors that confer survival advantage in early years may protect against neurodegeneration or delay its clinical manifestations. According to this model, current dementia frequency in developing countries would correlate inversely with infant mortality at the time that those at risk were born. In 1948, infant mortality in the UK was 38/1000 live births compared with 207 in Nigeria, 195 in China and 190/1000 in India. Infant mortality in Latin America varied from 40/1000 in Cuba, to 70 in Argentina, 81 in the Dominican Republic, 98 in Venezuela, 102 in Mexico, 109 in Peru and 135/1000 in Brazil. [38]. It seems plausible that as patterns of morbidity and mortality converge with those of the developed west, then dementia prevalence will do likewise [39].

### Dementia aetiology

#### Dementia, cardiovascular risk factors and cardiovascular disease

Recent research suggests that vascular disease predisposes to AD as well as to vascular dementia [40]. In short [41-43] and longer latency [44,45] incidence studies, smoking increases the risk for Alzheimer's disease. Inverse associations from case-control studies are thought to be explained by prevalence-incidence bias, with smoking influencing dementia-specific mortality [46]. Diabetes is also a risk factor [47], and in longer term cohort studies, midlife hypertension [48,49] and hypercholesterolemia [49] are associated with AD onset in later life. Aggregated cardiovascular risk indices incorporating hypertension, diabetes, hypercholesterolaemia and smoking incrementally increase risk for dementia incidence whether exposure is measured in midlife [45] or a few years before dementia onset [43]. Recent studies report associations between metabolic syndrome and incident cognitive decline [50], and insulin resistance and impaired executive function [51], but the role of the metabolic syndrome is as yet little explored. Despite occasional negative findings from large prospective studies [52,53], the accumulated evidence for a causal role for CVRF and CVD in the aetiology of dementia and AD is very strong. This has led to the hypothesis that atherosclerosis and AD are convergent disease processes [54], with some common pathophysiological and aetiologic factors (APOE e4 polymorphism, hypercholesterolaemia, hypertension, hyperhomocysteinaemia, diabetes, metabolic syndrome, smoking, systemic inflammation, increased fat intake and obesity).

#### Nutritional factors

Our review of the literature suggests two areas, of particular relevance to LAMICs, needing more research.

a) Research on micronutrients and dementia in developed countries has focussed upon antioxidants [55] with less attention towards deficiencies in vitamin B_12 _and folate, which result in hyperhomocysteinaemia; only four small incidence studies [56-59], all underpowered and with inconsistent findings. Two out of three studies testing for an effect of folate were positive [56,57], in one case independent of homocysteine [56]. B_12 _was associated in only one out of four studies [57].

b) Overt hypothyroidism is a potentially reversible cause of dementia. Sub-clinical hypothyroidism (raised TSH levels with normal T4) is more prevalent, affecting up to 20% of older people in developed countries. It is associated with elevated total cholesterol and progression to overt hypothyroid disease, possibly also CVD [60]. It was strongly associated with risk for dementia in one cross-sectional study [61]. Conversely, a small incidence study reported a strong association with sub-clinical hyperthyroidism (low TSH) [62].

### Ethnic differences and research in admixed populations

Previous research indicates strong and consistent ethnic differences in the incidence of dementia and stroke, in shared risk factors for atherosclerosis and dementia (lipid metabolism, hypertension, metabolic syndrome, APOE genotype), and in risk associations between certain of these risk factors and these outcomes.

#### Dementia

The age-specific prevalence [25] and incidence[26] of dementia in Nigeria are both very low. A further notable finding is the apparent lack of an association between APOE genotype and dementia [63], confirmed in Kenya [64]. Those with African ancestry tend to have a higher prevalence of APOE e4, but African Americans, other populations of west African ancestry, and Hispanics, all show weak and inconsistent associations with AD [65]. There is a robust association between APOE genotype and AD in Europeans and south Asians [66].

#### Stroke, heart disease and CVD risk factors

In the South London Stroke study [67] age and sex adjusted stroke incidence was higher among black (African Caribbean) residents, with a RR of 2.2 (1.8–2.8). In the USA incidence was higher among African Americans and Hispanics [68,69]. These differences may be partly explained by elevated blood pressure levels [70]. In the North Manhattan Stroke Project, both relative risks and population attributable fractions varied across white, black and Hispanic ethnic groups for the major stroke risk factors [71]. Conversely, African Caribbean migrants in the UK have a low risk of heart disease despite a high prevalence of metabolic syndrome, an effect attributed to a low prevalence of smoking and low triglyceride levels [70].

#### Admixture proportions

Measurement of individual admixture proportions (the proportions of the individual's genome that are of African, European, and Native American ancestry) is important for three reasons.

1. Studying the relationship of disease risk to admixture proportions is the most direct way to distinguish genetic from environmental explanations for ethnic variation in disease risk [72].

2. Such relationships will confound studies of any other genetic or environmental risk factors – "hidden population stratification". Measurement of the confounder allows us to control for it by standard methods.

3. Genes underlying ethnic variation in disease risk may be localized by admixture mapping, exploiting information about linkage generated by admixture [73].

## Aims of the 10/66 population-based research programme

### Primary objectives

1. To create a publicly accessible data archive from the 10/66 Dementia Research Group's cross-sectional population-based surveys including over 17,000 older people from six countries in Latin America and the Caribbean, India, China and Nigeria.

2. To use these data for comparative descriptive analyses of dementia prevalence, and its associated impact including: the economic costs of illness in each centre comprising indirect (informal care) costs, lost earnings and direct costs (health and social care and medication); the relative independent contribution of dementia and other major non-communicable disorders to direct and indirect costs, disability, dependency and caregiver strain.

3. To model dementia prevalence, examining the effects of age, education, literacy and SES, and at population level of urban vs. rural setting, historical levels of infant mortality, economic development, and industrialization.

4. To conduct a 2.5 to three year incidence phase follow-up of the baseline sample. The incidence phase will exploit the particularly rich baseline cross-sectional data in the six Latin American countries (Cuba, Dominican Republic, Venezuela, Mexico, Peru and Argentina) and in China, aiming:

5. To estimate the annual incidence rate of dementia and its subtypes, by age group, education and centre, and to investigate risk factors for incident dementia and AD, testing the following specific hypotheses: a) cardiovascular risk factors (diabetes, hypertension, large waist circumference, high waist hip ratio, hypercholesterolaemia, hypertriglyceridaemia, metabolic syndrome and smoking) and stroke increase the risk for incident dementia and AD, after controlling for confounding effects. Associations will be quantified as relative risks and population attributable fractions; b) micronutrient deficiencies (vitamin B_12 _and folate), and anaemia increase the risk for incident dementia and AD, and mediate any effect on these outcomes of overall nutritional deficiency; c) sub-clinical hypothyroidism is associated with increased risk of dementia and AD; d) incident dementia and AD are inversely associated with the proportion of the genome that is of African ancestry, and the association between APOE e4 genotype and incident dementia and AD is modified by admixture proportions – weaker in those with least African ancestry.

6. To confirm the predictive validity of the survey dementia diagnoses (DSM IV and 'the 10/66 dementia algorithm') and Mild Cognitive Impairment (MCI) through three year follow-up of all dementia and MCI cases. This will include a longitudinal study of evolving care arrangements and caregiver strain.

### Secondary objectives

#### 1. Mortality

a) to estimate annual all-cause and cause-specific mortality in each incidence centre; b) To investigate the independent effects upon all-cause and cardiovascular mortality of education, socio-economic status, cardiovascular risk factors (CVRF), prevalent cardiovascular disease (CVD), dementia, cognitive impairment, and depression

#### 2. Stroke

a) to estimate, in the same centres the annual attack rates for stroke; b) to compare the risk factor profile for incident stroke (relative risks and population attributable risk fractions) between centres and within centres between men and women and better and less well educated participants.

#### 3. Depression

To study the social aetiology of late-life depression cross-sectionally in all ten centres, and prospectively in the incidence centres including the effects of relative and absolute poverty, ill health and disability, nutritional status, social support, life events, marital circumstances and living arrangements.

## Methods/design

### The prevalence survey

#### Study design

Cross-sectional comprehensive one phase surveys have been conducted of all residents aged 65 and over of geographically defined catchment areas in each centre with a sample size of between 1000 and 3000 (generally 2000) in each of the ten countries (see Table 1). Each of the studies uses the same core minimum data set with cross-culturally validated assessments (dementia diagnosis and subtypes, mental disorders, physical health, anthropometry, demographics, extensive non communicable disease risk factor questionnaires, disability/functioning, health service utilisation, care arrangements and caregiver strain). The net result will be a unique resource of directly comparable data, comprising 19,000 older adults from three continents. DNA will be available from seven of the ten countries. Fasting blood samples have been taken at baseline from participants in seven countries – Brazil, Cuba, Dominican Republic, Venezuela, Mexico, Peru and Argentina, and analysed for full blood count and differential, glucose, cholesterol, triglyceride and albumin. Frozen serum has been saved for further biochemical analyses.

**Table 1:** Overview of 10/66 population based surveys

Country *(PI*)	Catchment area	Target sample	Interviews completed	Response %	DNA	Fasting blood sample	Start/finish
Cuba *(Llibre*)	Havana (urban)	2100	2100	94%	Yes	Yes	6/03 12/05
	Matanzas (urban)	900	900				
Brazil *(Menezes*)	Sao Paulo (urban)	2000	2000	93%	Yes	Yes	5/03 7/05
Dominican Rep. *(Acosta*)	Santo Domingo (urban)	2000	2000	95%	Blood (EDTA at -20 C)	Yes	11/03 12/05
Venezuela *(Salas*)	Caracas (urban)	2000	2026	79.6%	Yes	Yes	9/04 6/06
Mexico *(Sosa*)	Mexico city (urban)	1000	1000	85.1%	Blotting paper	Yes	1/06 10/06
	Morelos/Higalgo (rural)	1500	1000				
Peru *(Guerra*)	Lima (urban)	1500	1431	60%	Blotting paper	Yes	4/05 6/06
	Canete (rural)	500	569				
Argentina *(Arizaga*)	Buenos Aires (urban)	1200	0	incomplete	Yes	Yes	4/05 6/06
	Canuelas (rural)	800	800				
China *(Li/Huang*)	Xicheng (Beijing, urban)	1000	1000	83%	No	No	3/04 9/05
	Daxin (rural)	1000	1001				
India *(Krishnamoorthy*)	Chennai (urban)	1000	1005	72%	No	No	2/04 12/05
India *(KS Jacob*)	Vellore (rural)	1000	1000	98%	No	No	3/04 8/05
Nigeria (*Richard Ukawa*)	Anambra (rural)	1000	500	incomplete	No	No	
9 countries	13 catchment areas	20,000	17725				

#### Settings

Ten low and middle income countries (India, China, Nigeria, Cuba, Dominican Republic, Brazil, Venezuela, Mexico, Peru and Argentina). China, India, Peru and Mexico recruited from separate urban and rural catchment areas; the other centres included urban catchment areas only.

#### Catchment areas and sample registration

For urban catchment areas predominantly middle-class or professional areas with high-income earners were avoided. Rural catchment areas were defined by low population density, and traditional agrarian lifestyle. Catchment area boundaries were precisely defined. Mapping was carried out to identify and locate all households, which were allocated household IDs. Households were enumerated to identify possible eligibles (aged 65 and over). Age (and therefore eligibility) was formally determined on revisit for interview. For each household the genders and ages of all usual residents was recorded with the names of those aged 65 years or over on the census date. Household and participant details were stored in secure databases. These contained names, addresses, ID numbers, and contact details for neighbours, key informants and friends to facilitate tracing for potential follow-up. Precision calculations indicated that an overall sample of 2,000 would allow estimation of a typical dementia prevalence of 4.5% with a precision of +/- 0.9%. Rural and urban samples of 1,000 each will allow estimation of the same prevalence with a precision of +/- 1.2%.

#### Preparation

All centres had already been extensively trained in the main diagnostic assessments (see below) for the purpose of the dementia diagnostic pilot study [5]. Two further one week project planning meetings were held, in London for the PIs from Cuba, Brazil, DR, China and India and in Caracas for the Venezuelan, Mexican, Peruvian and Argentinean teams. One day follow-up meetings were held for all PIs in Barcelona and Dominican Republic. A standardized operating procedures manual covers every aspect of the training and field procedures.

#### Interviews and measures

All assessments were carefully translated into the relevant local languages (Ibero-American Spanish, Brazilian Portuguese, Tamil and Mandarin). Interviews were generally carried out in participants' own homes. This was a comprehensive one phase survey – all participants receive the full assessment, lasting approximately 2–3 hours.

##### 1) Outcome – The diagnosis of dementia

Our previously published 10/66 dementia diagnosis algorithm [5] requires

(i) A structured clinical mental state interview, the Geriatric Mental State, which applies a computer algorithm (AGECAT)[74], identifying organicity (probable dementia), depression, anxiety and psychosis and,

(ii) A cognitive test battery comprising a) the Community Screening Instrument for Dementia (CSI'D') COGSCORE [75] (incorporating the CERAD animal naming verbal fluency task), and b) the modified CERAD 10 word list learning task with delayed recall [76] and

(iii) An informant interview the CSI'D' RELSCORE [75], for evidence of cognitive and functional decline

(iv) an extended informant interview, the History and Aetiology Schedule – Dementia Diagnosis and Subtype (HAS-DDS), a modification of the earlier HAS [77], providing more detailed information on onset and course of a possible dementia syndrome

v) The NEUROEX, a brief fully structured neurological assessment with objectified quantifiable measures of lateralising signs, parkinsonism, ataxia, apraxia and primitive 'release' reflexes [78,79].

(vi) Behavioural and Psychological symptoms of dementia (BPSD); assessed using an informant questionnaire, the Neuropsychiatric Inventory (NPI-Q) [80].

Final dementia diagnoses is made in two ways. The main dementia outcome is defined as those scoring above a cutpoint of predicted probability of DSM IV Dementia syndrome [81] from the logistic regression equation developed in the 10/66 international pilot study, using coefficients from the GMS, CSI-D and 10 word list learning tasks [5]. The second approach involves the direct application of research diagnostic criteria for DSM IV and for the following dementia subtype diagnoses; NINCDS-ADRDA Alzheimer's disease criteria [82], NINDS-AIREN vascular dementia criteria [83], and Lewy Body Dementia [84]. The clinical assessment identifies other prevalent conditions relevant to the differential diagnosis of dementia and dementia sub-type: psychosis, depression, anxiety disorder, alcoholism, epilepsy and stroke. Circularity in further validation of these diagnoses can only be avoided by focusing upon their predictive validity. Thus, a diagnosis of dementia should lead to further cognitive and functional decline, and not recovery.

##### 2) Blood sample protocol

Fasting blood samples were sought from all participants in all centres other than India, China and Nigeria, necessitating revisits for phlebotomy first thing in the morning. 15 mls of blood was drawn and distributed as follows; one 5 ml plastic EDTA for haematology and DNA extraction or storage, one 2.5–3 ml Fluoride Oxalate bottle for glucose and the remainder (approx 8 ml) to a clot bottle for biochemistry. Samples were transferred immediately to the laboratory for haematological and biochemical analysis (see below). Serum obtained from centrifuging the clot sample was divided into aliquots and fresh frozen at -20 C. For the DNA collection, in Cuba and Venezuela the EDTA samples are processed and DNA extracted immediately. In Dominican Republic the EDTA sample is frozen at -20 C. In Peru, Argentina and Mexico blood is collected onto FTA Elute (Isocode) cards. Each 3 mm punch yields 20 × 50 ul PCR reactions (100 × 10 ul) so one card set generates 640 × 50 ul PCR reactions.

##### 3) Genotyping

DNA will be extracted and stored from all seven centres. APOE genotyping will be conducted on all samples, and 60 SNPs informative for African/European ancestry admixture will be genotyped from centres where admixture is relevant (Cuba, Dominican Republic, Mexico, Peru and Venezuela). The 60 SNPs will be chosen from the panel assembled by Dr Mark Shriver at Penn State and Mike Smith at NCI [85]. With 60 SNPs that have average 40% information content for ancestry, we shall be able to estimate three-way individual admixture proportions with a standard error of less than 0.1.

##### 4) Principal exposures – environmental

This information was elicited from participants; with informants also interviewed for those with communication difficulties arising from dementia, severe mental illness, deafness or mutism.

###### 4.1 Sociodemographic status

a) age, living circumstances (complete list of coresidents with ages and relationships), marital status, education, literacy, religion affiliation and practice, community social activity, social support, social network

b) Socio-economic status – best occupation (self and spouse), current occupational status, income and sources of income, household assets index, food insecurity

c) Migration status – Rural or urban residence across the life course. Age when moved,

###### 4.2 Health status

a) self reported global health,

b) self-reported diagnoses (stroke, diabetes, hypertension, heart disease, hypercholesterolaemia, TB, malaria, and cystercicosis) and treatments for these conditions

c) a self-reported list of 12 commonly occurring physical impairments [86];

d) activity limitation and participation restriction measured by the WHO-DAS II [87], specifically developed by the WHO as a culture-fair assessment tool for use in cross-cultural comparative epidemiological and health services research.

e) direct physical assessments – pulse rate, systolic and diastolic resting blood pressure (average of two, sitting and standing), waist circumference, waist/hip ratio, walking test (5 metres walk, turn and return – timed and paces counted).

f) Reproductive status (for women) – menarche, menopause, reproductive period, number of children

###### 4.3 Biological assessments

a) Physical assessments – pulse rate, systolic and diastolic resting blood pressure (average of two, sitting and standing), waist circumference, waist/hip ratio, leg length, height, skull circumference, walking test (5 metres walk, turn and return – timed and paces counted).

b) Haematological tests; full blood count (haemoglobin, haematocrit, differential, MCV, MCH, MCHC).

c) Biochemical tests; fasting glucose, fasting total cholesterol and sub-fractions, triglyceride, albumin, total protein.

These data collected allow us to identify metabolic syndrome according to the criteria proposed by the Third Report of the National Cholesterol Education Program (NCEP – ATP III) : presence of three or more of the following; 1. Central obesity as measured by waist circumference: Men > 40 inches, Women > 35 inches. 2. Fasting triglycerides >= 150 mg/dL. 3. HDL cholesterol: Men < 40 mg/dL, Women< 50 mg/dL. 4. Blood pressure >= 130/85 mmHg. 5. Fasting glucose >= 110 mg/dL

###### 4.4 Risk exposures

a) specific dementia risk factors – head injury with loss of consciousness, family history of dementia, previous depression, leg length, height, skull circumference,

b) Lifestyle and cardiovascular risk factors – alcohol use (volume and frequency currently and before the age of 60), lifetime smoking (never, ever and current smokers and pack year calculation), diet (intake of fish, meat and fruit and vegetables; food insecurity), exercise and activity levels now and in earlier life.

##### 5) Measures of care arrangements, and impact of providing care on caregivers

These measures have been refined and validated in the 10/66 group's pilot studies[9,12].

a) Economic impact was assessed using the Client Service Receipt Inventory [88], a comprehensive assessment of direct and indirect economic costs for mental health services, adapted for use in the developing world. It elicits information on type and cost of accommodation, income (from all sources) for the person with dementia and the principal caregiver, the occupation of the caregiver, the extent to which the caregiver had cut back on or stopped work in order to provide care, unpaid care provided by family or others in the community, paid care inputs and their costs, and the use (and associated costs) of a variety of health care services; hospital services (inpatient and outpatient); government community health services (general practitioner, nurse, community health worker, other health worker); private doctor; dentist; time and duration of visits, out of pocket costs for the consultations and medications, time taken to travel, cost of travel.

b) Practical impact – contact time between caregiver and cared for person [89], whether or not care was being provided. Time spent by the caregiver in the last 24 hours in specific caregiving activities [90]; communicating, using transport, dressing, eating, looking after one's appearance, and supervising,

c) Caregiver perceived strain – the Zarit Burden Interview (ZBI) [91-93] with 22 items that assess the caregiver's appraisal of the impact their involvement has had on their lives.

d) Caregiver mental health (the Self Reporting Questionnaire 20) [94].

e) Behavioural and Psychological symptoms of dementia; the Neuropsychiatric Inventory (NPI-Q)[80]

#### Resources/training/quality control

Each centre had a project coordinator and 4–10 interviewers. These were generally lay graduates, although Cuba and China used medical doctors. All researchers were rigorously trained in a) study protocol and procedures, b) standard structured interviewing techniques, c) a specific two day training for the Geriatric Mental State structured clinical mental status assessment and the neurological/physical examination. MP had trained the project PIs and video training materials were provided for local centres. Field interviews were regularly checked and supervised.

#### Data management

In Cuba, all data was collected directly onto laptop computers using computerized Spanish questionnaires driven by EpiData (version 2.0) software. These questionnaires, developed by the 10/66 group incorporate conditional skips, and interactive checking of data consistency. In other Hispanic centres, data was collected onto paper and double data entered onto the same EpiData files. Chinese and Indian centres used English language data entry files. Identical EpiData files in all centres facilitates data processing, data merging and archiving. Data is extracted into SPSS, and all processing (cleaning, processing of derived variables and running of 10/66, DSMIV dementia and other diagnostic algorithms) is carried out using SPSS batch files. The end result is a cleaned, processed and labelled data set that can be exported into other statistical programs for further analysis.

#### Ethical issues

Participants were recruited following informed signed consent. Persons with dementia who lack capacity for consent were recruited on the basis of a relative's signed agreement. Illiterate persons were read the information sheet and consent form, and invited to express their consent verbally, which was witnessed. Participants were invited to make a gift of genetic material (blood) to the local research institutions who act as custodians in whom are vested full rights for scientific and commercial exploitation, subject only to limitations upon the phenotypes to be investigated. Studies were approved by local ethical committees as well as by the ethical committee of the Institute of Psychiatry, King's college London.

### Incidence phase

#### Tracing participants

Response rates for the baseline surveys were generally high, ranging from 74 to 98% (see Table 1). For the follow-up, which was pre-planned at baseline, we have collected the names, addresses and telephone numbers of three non co-resident local relatives, friends or neighbours who can be contacted to help trace the participant if they are not at their original address. The population in each of the settings where we are working is stable. Apart from a likely 6% per annum attrition from mortality, we anticipate a very high follow-up rate, in excess of that typically achieved in cohort studies in developed countries. All methods, procedures and logistics are well established from the 10/66 baseline surveys.

#### Protocol

The interviews for the incidence phase largely involve a repeat of the one phase dementia diagnostic protocol previously described, with the aim of identifying incident dementia. We will modify the DSMIV algorithm to incorporate cognitive test and informant data from baseline into the direct determination of cognitive and functional decline. We will also reassess risk exposures that may have changed over the three years since baseline. All participants will receive the same comprehensive assessment, very similar to that administered in the baseline survey, regardless of their baseline status. Those with dementia at baseline will have been at risk for stroke, and we will repeat dementia assessments to assess course and outcome and to validate baseline diagnoses. We will also review care arrangements, dependency and indices of caregiver strain. Interviewers will be blinded to baseline diagnostic status.

### Interviews and data collection

#### Mortality

For those who died between baseline and follow-up (information ascertained on follow-up visit) we will complete a verbal autopsy interview with a co-resident, relative or other person well-placed to know the circumstances of death, using methods developed and validated by the 'Million Deaths' project for use in India [95,96] to identify underlying cause according to ICD10 criteria. The interview takes 30–45 minutes using structured questions and an open-ended narrative, with a symptom list to assist attribution. Cause of death is allocated by the consensus judgement of two physicians. The approach is valid for use up to three years post-mortem. We will also attempt to ascertain whether the participant had an onset of dementia before death, using the standard 10/66 informant assessment.

#### Stroke

We also include a two phase clinical protocol designed to identify incident stroke. All those claiming (by participant or informant report) to have experienced a stroke in the interval between assessments, and all those with suggestive neurological signs (asymmetric long tract signs, dysphasia, marked gait disturbance) not apparent at baseline will be offered physician assessment including physical examination, clinical history, and examination of clinical notes and investigations where available ('cold pursuit'). We will then seek consensus diagnosis from two local independent experts. Stroke diagnosis (ARIC criteria [97]) require

a) evidence of sudden or rapid onset of neurological symptoms

b) lasting for more than 24 hours or leading to death,

c) in the absence of evidence for a nonstroke cause (brain trauma, neoplasm, coma attributable to metabolic disorders or disorders of fluid or electrolyte balance, CNS vasculitis or infection, and peripheral neuropathy).

In our baseline data set 96% of those who reported a stroke had a clinical diagnosis, 81% from a specialist. However, neuroimaging will not routinely be available, and we cannot be confident that we will have enough information to attribute stroke sub-type with confidence. Silent brain infarctions (picked up by neuroimaging without clinical symptoms and signs) will not be included.

#### New retrospective exposure data (not collected at baseline)

Number of remaining teeth, use and quality of dentures

Use of lipid-lowering agents

Calf circumference

Weight (using digital scales)

Grip strength

Interviewer assessment of mobility (bedbound/chairbound/housebound/limited mobility outside/unrestricted mobility

#### New biochemical assays

Fresh frozen serum from baseline collections are available in Cuba, DR, Venezuela, Peru, Argentina and Mexico. We will use these to investigate associations between micronutrient deficiency and dementia/AD. We will use a nested incident case control design whereby for each incident case, we shall select three controls at random from among those matching for age, gender and education, who were free of dementia at the time of onset for the case. Assuming 219 incident cases in these six centres (see below) this will imply 876 samples to be assayed for Vitamin B12, folate, TSH and T4. Routine assays will be carried out by local laboratories, but procedures will be standardised as far as possible. Fresh frozen serum stored at -20 C is appropriate for these analyses.

### Analyses

#### Description of incidence

Person-years at risk will be calculated as the interval between baseline and follow-up assessment, or the estimated time of onset of dementia, or the time of death, whichever occurs sooner. Age-specific incidence (with Poisson standard errors and 95% confidence intervals) will be estimated for each country, by gender and age in 5-year bands by dividing number of cases by number of person-years contributed in each age band. Dementia onset is assumed to be the midpoint between the last date when known to be dementia free and the first date of dementia diagnosis (either by survey ascertainment or clinical information). A similar procedure will be used to calculate stroke attack rates.

#### Hypothesis testing

We will use Cox's proportional hazards regression throughout to estimate risk associations. Informative censoring may occur through the competing risks of participants dying and becoming lost to follow-up in the interval between assessments. This problem is only rarely addressed in dementia cohort studies [53]. We will do so firstly by verbal autopsy interviews with key informants for all deceased persons, and secondly (in a sensitivity analysis) by using proportional subdistribution hazards regression [98] to account for informative censoring through explicit analysis of competing risks.

The ADMIXMAP program [99] will be used to model genetic admixture. In two-step analysis, estimates of individual admixture generated by ADMIXMAP are simply plugged into standard programs for statistical analysis. One-step analysis fits regression models for the effect of individual admixture and other risk factors (genetic or environmental) on disease risk. This allows for the uncertainty in estimation of individual admixture from the marker data, eliminating residual confounding by population stratification that may occur in a two-stage analysis using a small number of markers, and allows us to model haplotypes given unphased genotype data. For this study, ADMIXMAP will be extended to support Cox regression (regression algorithms are based on a GLM approach). We shall test for

1) effects of individual admixture on dementia, AD and other outcomes

2) effects of individual admixture on the slope of relationship of APOE to dementia.

3) effects of other environmental factors or genetic polymorphisms on dementia, again controlling for population stratification.

#### Power and sample size considerations

Power estimations were carried out using Stata 8.2 ssmenu command [100] for sample size and power calculations in complex studies with failure time outcomes (table 2). Pooling the data across the six Latin American centres yields the following power (Table 3) for identification of the following effect sizes for associations with incident dementia and stroke, assuming the following prevalences of risk exposure.

**Table 2:** Incident dementia, stroke and all-cause mortality in centres participating in the follow-up study

Country	Baseline sample	Dementia at baseline	Free of dementia at baseline	Available for FU interview^1^	Person years	Deaths	Incident dementia^2^	Incident strokes
Cuba	3000	330	2670	1994	6730	509	55	45
DR	2000	220	1780	1329	4488	339	37	30
Venezuela	2000	200	1800	1345	4490	339	37	30
Mexico	2000	200	1800	1381	2906	295	30	25
Peru	2000	200	1800	1381	2906	295	30	25
Argentina	2000	200	1800	1381	2906	295	30	25
Sub-total^3^	13000	1350	11650	8811	24426	2072	219	180
China	2162	137	2025	1513	5105	366	42	30
TOTAL	15000	1300	11700	10324	29531	2438	261	210

1. assuming 6% annual mortality (GBD 2002 AMRO B mortality data, 65 and over), and 15% non-response/non-traceability.

2. annual incidence rate of 0.0092 for all those aged 65 and over, from consensus prevalence estimates for Latin America [19], using DISMOD to derive incidence from prevalence and survival.

3. These centres included fasting blood samples, which are lacking in China

**Table 3:** Power to detect given effect sizes (HRs) for associations between exposures with a prevalence of 10% and 20%, for the outcomes of dementia and stroke.

Minimum Hazard Ratio to be detected	Power for detecting association with 10% exposure prevalence		Power for detecting an association with 20% exposure prevalence	
Outcome^1,2^	Dementia	Stroke	Dementia	Stroke

2.5	100%	99.8%	100%	100%
2.0	96.4%	90.9%	99.8%	98.8%
1.8	86.0%	75.8%	97.5%	92.8%
1.5	48.8%	39.3%	71.0%	59.5%

1. The power to detect associations with all-cause mortality approximates to 100% in each of these cells

2. As approximately 70% of dementia cases are categorised as AD, the power of detecting given associations with this outcome is very similar to that for stroke.

For the nested incident case-control study we estimate that 219 cases and 657 matched controls will be available. Given the matching, all analyses will be carried out using conditional logistic regression (Stata 8.2). For exposure prevalences ranging from 5% (lowest realistic estimate of prevalence for folate deficiency and subclinical hypothyroidism) to 40% (likely prevalence of vitamin B_12 _deficiency) such a comparison will have good (80%) to excellent (90%) power for detecting policy-relevant effects (Table 4).

**Table 4:** Minimum detectable effect size (OR) for matched incident case-control comparison

**Exposure Prevalence Power**	**5%**	**10%**	**15%**	**40%**
80%	2.4	2.0	1.8	1.6
90%	2.7	2.2	2.0	1.7

### The casefinder study

After one day of training on the clinical characteristics, presentation and course of dementia, community key informants will generate, from their knowledge of the local population names of those living in the catchment areas to be surveyed, who they think may be possibly or probably suffering from dementia. Key informants may be community health workers, social welfare officers, community activists or other volunteers depending upon the local health system. The aim is to identify where possible the cadre of workers with a community outreach role and some consequent knowledge of the circumstances of all or many of the local inhabitants. These assessments will then be validated against the comprehensive structured clinical diagnostic assessment from the subsequent cross-sectional survey. The sensitivity, false positive rate, and positive and negative predictive values of the dementia case-finding procedure will be estimated with respect to the criterion of the structured clinical research diagnosis. The characteristics of those with research dementia diagnoses who were and were not nominated by community key informants will be compared to identify factors predicting failure of detection. Factors to be assessed include age, gender, marital status, living circumstances, socio-economic status, rural or urban residence, recent use of health services, severity of dementia, subtype of dementia, co-morbidity, associated behavioural problems, time spent caregiving, caregiver strain and mental health status. A sample of 2,000 in each centre will provide approximately 90 cases of dementia allowing sensitivity to be estimated with a precision of +/- 7%, and a difference in sensitivity between (for example) rural and urban areas of 30% (80% vs 50%) to be detected with 95% confidence and 80% power.

### The 10/66 caregiver education and training intervention

The 10/66 intervention was developed in India (for use by multi-purpose health workers), but with input from the wider 10/66 group including developed country experts. It targets the main carer, but includes members of the extended family. The aim is to provide basic education about dementia and specific training on managing problem behaviours. The three simple, manualised modules are delivered over five, weekly, half hour sessions.

#### Module 1. Assessment – 1 session

a) Cognitive/functional impairment b) Carer's knowledge and understanding of dementia. c) Care arrangements (Who are the family members? Who lives with the person with dementia? How do they assist the main carer? Which behavioural problems present most difficulties? How burdened do they feel?).

#### Module 2. Basic education – 2 sessions

a) general introduction to the illness. b) what to expect in the future c) What causes/does not cause dementia? d) locally available care and treatment.

#### Module 3. Training on problem behaviours – 2 sessions

Problem behaviours identified in the assessment are addressed (personal hygiene, dressing, incontinence, repeated questioning, clinging, aggression, wandering, apathy). After extensive piloting the intervention is now supported by a structured, manualised two day training program for MPHWs comprising a) basic knowledge about dementia, b) assessment skills, c) basic counselling skills and d) role playing of intervention scenarios.

In Argentina, Mexico, Peru, Venezuela, Russia, and China, we are carrying out a formal randomised controlled trial; all people with dementia identified in the population-based survey (and their carers) will be offered randomisation to receive the caregiver training and education intervention either a) immediately or b) after 6 months, that is after the final outcome assessments will already have been completed. The inclusion criterion is a 10/66 algorithm diagnosis of dementia. Exclusion criteria are serious intercurrent illness in the person with dementia, or absence of family caregivers. Consent is obtained from the person with dementia, where possible, and their principal caregiver. The principal caregiver is the family member or close friend who is most involved in providing and/or organising care for the person with dementia. Paid caregivers and/or other family caregivers may participate in the intervention but are not subject to the outcome assessment. In the pre-randomisation visit, a research worker completes the baseline assessments (see below), explains the trial and seeks informed consent from the person with dementia and the caregiver. Randomisation is carried out in London, and the codes transmitted to the local 10/66 centre by fax or e-mail. Randomisation is by permuted block to ensure in each centre an even distribution of baseline caregiver strain. The intervention in all centres will be evaluated in terms of uptake rates, completion rates and satisfaction rates (among caregivers), together with before (baseline) and after (six months) intention to treat comparisons of standard assessments. Principal outcomes related to the caregiver are caregiver role strain (Zarit caregiver burden interview), psychological distress (SRQ 20), and quality of Life (the 17 item WHO-QoL Bref) [101]. Principal outcomes related to the person with dementia are behavioural and psychological symptoms of dementia (Neuropsychiatric Inventory – NPI-Q) [80] and quality of life (DEMQoL) [102].

The clinician (if any) responsible for clinical care of the person with dementia will be blind to the intervention. They may organise any supplementary care that they feel is indicated. The assessor, assessing outcome after the intervention will be blind to the intervention. The family, and the assessor will be asked to do everything they can to maintain blindness. Primary endpoint analysis will be according to intention to treat with last observation carried forward for missing data. Failures of randomization would be adjusted for as factors or covariates, using generalized linear modelling. In each centre, with 30 families randomised to the control and intervention arms, the study is powered to detect moderate effect sizes (0.8 or greater for change scores on continuously distributed outcomes) associated with the intervention, at 80% power and 95% confidence. Data from the different centres would be subject to meta-analysis. (ISRCTN41039907; ISRCTN41062011; ISRCTN95135433; ISRCTN66355402; ISRCTN93378627; ISRCTN94921815).

## Discussion

### The contribution of the 10/66 studies

Despite some excellent studies, dementia in LAMIC remains under-researched, particularly given its large, growing public health and societal impact. There are inequities in the increasing attention directed towards NCDs in LAMIC. Relatively little research, policy or practice is directed towards older adults. Premature mortality is given more attention than living with disability. According to the 2003 World Health Report Global Burden of Disease estimates, dementia contributed 11.2% of all years lived with disability among people aged 60 and over; more than stroke (9.5%), musculoskeletal disorders (8.9%), cardiovascular disease (5.0%) and all forms of cancer (2.4%). Our programme of population-based studies in ten developing countries provides a powerful resource for comparative descriptive research of prevalence, incidence, impact and cost. The main aim of the incidence phase is to investigate aetiology (a particularly neglected area). Findings from the west (for example, cardiovascular risk factors as risk factors for dementia) may, or may not generalise. Both exposure prevalence and effect sizes may differ between regions because of population-specific genetics, culture (behaviour and lifestyles) and physical environment. Other exposures, for example micronutrient deficiency, are rare in richer countries but highly prevalent and potentially of great significance in Latin America and China. Population attributable fractions will assist in determining priorities for future health promotion and primary care prevention in these regions. While cardiovascular disease and its risk factors are also relatively little studied in Latin America, two new initiatives will soon contribute relevant data[103]; the PREVENCION study in Arequipa, Peru involves a comprehensive assessment of cardiovascular risk factors and outcomes among 1600 community residents aged 20–80, while the CARMELA study will provide similar data in younger samples (aged 25–64) from seven countries – Colombia, Argentina, Peru, Mexico, Ecuador, Chile and Venezuela. Our 10/66 population-based studies will neatly complement the CARMELA database by providing information from many of the same countries on those aged 65 and over.

### Methodological issues

Our one phase dementia diagnostic assessment has advantages over the two phase approach used in most previous dementia cohort studies [104]. Attrition is marked between the first and second phase [2]; participants with probable dementia are particularly likely to refuse, to move away or to die, leading to informative censoring. The problem is compounded when no random sample of screen negatives is selected for second phase assessment with the tacit assumption of perfect sensitivity for the screening measure [104-106].

The 10/66 dementia diagnosis has been carefully validated across all of the cultures, and in each of the centres in which the population research is proceeding. While sensitivity (94%) and specificity (97% in high education controls and 94% in low education controls), against the gold standard of a local clinician's DSM IV diagnosis were both excellent, the false positive rate which varied between 1% and 10% across regions and levels of education can be expected to result in a higher prevalence of 10/66 dementia, compared with that of DSM IV dementia. DSMIV criteria are recognized to be relatively restrictive. Impairment is required in memory and other specified domains of function. Each of these must have progressed, and led to social or occupational impairment. The deficits should not be explained by delirium or other mental disorder. The aim with DSMIV (and other similar diagnostic criteria) was to define a progressive and relatively pervasive disorder with thresholds set high to maximize reliability between raters and centres. Clinically relevant dementia may therefore be prevalent beyond the confines of the narrowly defined DSM IV criterion. One of the few population-based studies to examine this issue directly, the Canadian Study of Health and Aging [107] reported a prevalence of 20.9% for those aged 65 and over according to clinical consensus compared with 13.7% according to DSM IV criterion. Mild cases, confirmed by clinicians, were selectively excluded by the DSM IV criterion. None of the DSM IV criteria are specifically required by the 10/66 probabilistic algorithm, which simply requires a profile of cognitive impairment on formal testing, informant reports of cognitive and functional decline, and findings on clinical interview that are consistent with a high probability of being a case. While the DSMIV algorithm identifies clear cut, severe and pervasive dementia cases, the 10/66 algorithm may be more relevant to establishing the true population burden of the dementia syndrome.

The catchment area sampling strategy enables us to foster links within each local community, improving response and facilitating possible follow-up. Community sensitisation proved to be essential to ensure a good response to the surveys. Prevalence estimates and other descriptive elements may not generalize beyond these and similar communities, but this is unlikely to lead to bias in estimates of association.

### Dissemination

An action research program of this kind stands or falls upon its ability to inform and encourage policy development on the basis of the evidence accumulated through its activities. The 10/66 Group recently held a one week workshop at the Rockefeller Center in Bellagio to address the need to exploit fully the potential created by the 10/66 population based and intervention studies. Dissemination through a peer reviewed scientific journal is but one important element of this process. Our challenge is to use our findings to raise public awareness, stimulate local clinical training and practice, and influence social welfare and health care policy making at the national and international level. In these respects, our relationship with ADI (Alzheimer Disease International) is crucial. ADI is affiliated to the World Heath Organisation. The needs of people with dementia in developing countries is now a major priority for ADI, and our findings are disseminated on its website [3], at its conferences, and in its regular newsletters and World Alzheimer's Day Bulletins, distributed to its 76 member associations worldwide. In turn, the national Alzheimer's associations are able to use our materials for local publicity, and to influence national policy makers as part of their lobbying activities at government level. Other committed NGOs may have a key advocacy role to play, and we will need to work intersectorally to maximise dissemination and policy impact. Dementia is one of many health conditions in the developing world characterised by lack of awareness, stigma, limited help seeking, few services, and much unmet need. The evidence provided by research, disseminated actively by committed NGOs can be a powerful argument for change.

At the Bellagio meeting we devised a strategy for dissemination focusing upon: Identifying key stakeholders; designing and conducting local workshops; forging links with policymakers, understanding their preoccupations, and preparing policy briefings in each of the countries/regions where we are working; linking with the print and radio news media in developing countries, and understanding how its support may be elicited in raising awareness; exploiting links between 10/66 researchers and national Alzheimer's Associations; forming intersectoral links with other relevant NGOs e.g HelpAge International, Save the Children (cross-generational effects), Oxfam (economic impact of aged care, and poverty reduction)

All investigators in this programme are committed to establish a monitored public access fully anonymised file sharing archive, to maximize exploitation of the data resource. The Brazilian centre is making its own local arrangements. For all other centres, data will be forwarded to the coordinating centre for further cleaning and checking for compatibility with the uniform file format. The final version will be approved by the local centre, archived in a secure directory on the Institute of Psychiatry network, and shared with all centres participating in the archive. Proposals for multi-centre publications may be made by the coordinator, by centre PIs, or by external investigators. Access will not unreasonably be denied, and will be approved by a publications committee with two members from each centre. The 10/66 programme steering committee will give independent oversight. In the interests of transparency, all interviews, data entry files, algorithms, protocols, training materials and manuals can be downloaded from our intranet site[108]. Applications for use of the data should be sent to 1066drg@iop.kcl.ac.uk.

## List of abbreviations

AD – Alzheimer Disease

ADI – Alzheimer Disease International

AGECAT – The Automated Geriatric Examination for Computer Assisted Taxonomy

APOE – apolipoprotein E

ARIC criteria – Atherosclerosis Risk in Communities criteria

BPSD – Behavioural and Psychological Symptoms of Dementia

CERAD – Consortium to Establish a Registry for Alzheimer's Disease

CNS – Central Nervous System

CSI-D – Community Screening Interview for Dementia

CVD – cardio vascular disease

CVRF – Cardio vascular risk factors

DEMQoL – Dementia Quality of Life instrument

DR – Dominican Republic

1066 DRG – Dementia Research Group

DSM – Diagnostic and Statistical Manual of Mental Disorders

EDTA – ethylenediaminetetraacetic acid

GI – Gastro intestinal

GLM – Genera; Linear Model

GMS – Geriatric Mental State

HAS-DDS – History and Aetiology Schedule Dementia Diagnosis and Subtype

ICD 10 – International Classification of Diseases

IHD – Ischaemic heart disease

LAMIC – low and middle income countries

MCH – Mean Corpuscular Haemoglobin

MCHC – Mean Corpuscular Haemoglobin concentration

MCI – Mild Cognitive impairment

MCV – Mean corpuscular volume

MPHW – Multi purpose health worker

NCD – non-communicable diseases

NGO – non governmental organization

NINCDS-ADRDA – National Institute of Neurological and Communicative Disorders and Stroke and the Alzheimer Disease and Related Disorders Association Criteria for Alzheimer Disease

NINDS-AIREN – National Institute of Neurological Disorders and Stroke and Association Internationale pour la Recherché et l'Enseignement en Neurosciences

NPI-Q – Neuropsychiatric inventory

PI – principal investigator

RR – risk ratio

SNP – Single Nucleotide Polymorphism

SRQ – Self Report Questionnaire

T4 – Thyroxine

TB – Tuberculosis

TSH – Thyroid-Stimulating Hormone

WHO-DAS – World Health Organization – Disability assessment schedule

WHO-QoL Bref – World Health Organization – Quality of Life BREF

ZBI – Zarit Burden Interview

## Competing interests

The 10/66 Dementia Research Group works closely with Alzheimer's Disease International (ADI), the non-profit federation of 77 Alzheimer associations around the world. ADI is committed to strengthening Alzheimer associations worldwide, raising awareness regarding dementia and Alzheimer's Disease and advocating for more and better services for people with dementia and their caregivers. ADI is supported in part by grants from GlaxoSmithKline, Novartis, Lundbeck, Pfizer and Eisai.

## Authors' contributions

MP and CPF prepared the first draft. Other authors reviewed the manuscript, provided further contributions and suggestions. All of the authors worked collectively to develop the protocols and methods described in this paper. All authors read and approved the final manuscript.

## Pre-publication history

The pre-publication history for this paper can be accessed here:


